# Stercoral Perforation of Sigmoid Colon in Systemic Lupus Erythematosus: A Rare Cause of Peritonitis

**DOI:** 10.7759/cureus.9495

**Published:** 2020-07-31

**Authors:** Ejaz Latif, Shameel Musthafa, Aryan Ahmed, Ahmad Abu Amr

**Affiliations:** 1 General Surgery, Hamad Medical Corporation, Doha, QAT; 2 Acute Care Surgery, Hamad Medical Corporation, Doha, QAT

**Keywords:** stercoral perforation, fecal peritonitis, systemic lupus erythematosus, large bowel perforation, constipation, sigmoid perforation

## Abstract

Stercoral perforation (SP) is a rare cause of peritonitis. It is caused by pressure necrosis of the colonic wall by fecaloma. SP is a lethal condition that is associated with high morbidity and mortality, therefore early diagnosis and treatment are of paramount importance. Herein, we describe a case of SP in a systemic lupus erythematosus (SLE) patient.

A 44-year-old female, known case of SLE, presented with severe abdominal pain, fever, and hypotension. CT scan showed features of perforated sigmoid. The patient underwent exploratory laparotomy which revealed perforation of sigmoid, fecalomas in the peritoneal cavity, and colon loaded with fecal matter. The patient underwent Hartmann's operation with successful control of her intra-abdominal sepsis. Her postoperative course was complicated by SLE flare and wound dehiscence which was probably due to long term steroid use.

Even though SP is rare, it carries a worse prognosis especially if the patients are immunocompromised. The key to successfully manage such cases is early diagnosis, aggressive resuscitation, antibiotics, and prompt surgical intervention. A multidisciplinary approach is often helpful in such cases.

## Introduction

Stercoral perforation (SP) is one of the rare causes of colon perforation which is associated with a mortality of 32% [1]. Most published literature on SP credit James Berry for describing SP first in 1894, when he reported a case of sigmoid perforation, but he proposed that the mechanism of perforation was due to atony of the sigmoid leading to overdistension of the bowel and never used the term stercoral or stercoraceous [2]. However, after a thorough literature search and examination of journal archives, we found that the term was actually first used by J. J. O’Reilly in 1935 when he reported a classic case of “stercoraceous ulcer” perforation due to long-standing pressure from stool balls in the colon causing ischemia [3].

In the literature, there are less than 150 cases reported until 2018 [4]. Although intestinal perforation in systemic lupus erythematosus (SLE) patients are reported, we describe a unique case of SP in an SLE patient caused by fecaloma [4,5]. To the best of our knowledge, this is the first case reported in English medical literature. Our case also presented a challenge in the management as she was immunocompromised with a complicated postoperative course.

## Case presentation

A 48-year-old lady of Central African descent, a known case of SLE on steroids, presented to the ED with a history of abdominal pain for one day. The pain had a sudden onset, was of a sharp character, severe intensity, continuous and non-radiating. Initially, it was in the lower abdomen and then it became generalized. The pain was aggravated on movement and it was associated with spikes of fever, chills, and anorexia. She had a long history of constipation but she denied having nausea, vomiting, diarrhea, abdominal distension, melena, hematemesis, or bleeding per rectum. There was no history of recent trauma or falls. Her past medical history is significant for hypertension, hypothyroidism, and lupus nephritis. She was maintained on 5 mg prednisolone for the past 10 years, azathioprine, hydroxychloroquine, verapamil, irbesartan, and levothyroxine. She denied any prior surgeries, cardiac events, or palpitations.

Examination

On presentation, she was conscious, oriented, diaphoretic, in pain, and lying still in her bed. She had dry oral mucosa and her urine was concentrated indicating dehydration. Her blood pressure was 99/60 mmHg, pulse rate was 106/min and regular, respiratory rate was 36/min, the temperature was 39 degree Celsius, and her oxygen saturation was 92% on room air.

Abdominal examination revealed a generalized abdominal tenderness with marked tenderness, guarding, and rebound in the lower abdomen. There were no visible scars, skin changes, ecchymosis, or palpable masses. Digital rectal examination revealed mucoid stool and it was negative for blood, melena, or a mass.

Investigations

Her lab values (with normal range in parenthesis) at presentation showed hemoglobin 9.7 g/dL (12.0 to 15.0), WBC 2.8 x 103 per microliter (4.00 to 10.00), lactic acid 4.5 mmol/L (0.5 to 1.6), procalcitonin 25 ng/mL (0.0 to 0.5) showing severe sepsis, C-reactive protein 112 mg/L (0 to 5), pH 7.3 (7.35 to 7.45), bicarbonate 14.3 mmol/L (20 to 28) creatinine 93 micromoles/L (50 to 98), blood urea 11.6 mmol/L (2.5 to 6.7) and albumin 31 gm/L (35 to 50). Serum pancreatic amylase and lipase were normal. A bedside urine dipstick test was negative for nitrites or leukocyte esterase. As her septic markers were high and lactate was elevated, so we ordered a chest X-ray to look for gas under the diaphragm and CT scan with contrast to rule out mesenteric ischemia.

Imaging

**Figure 1:**
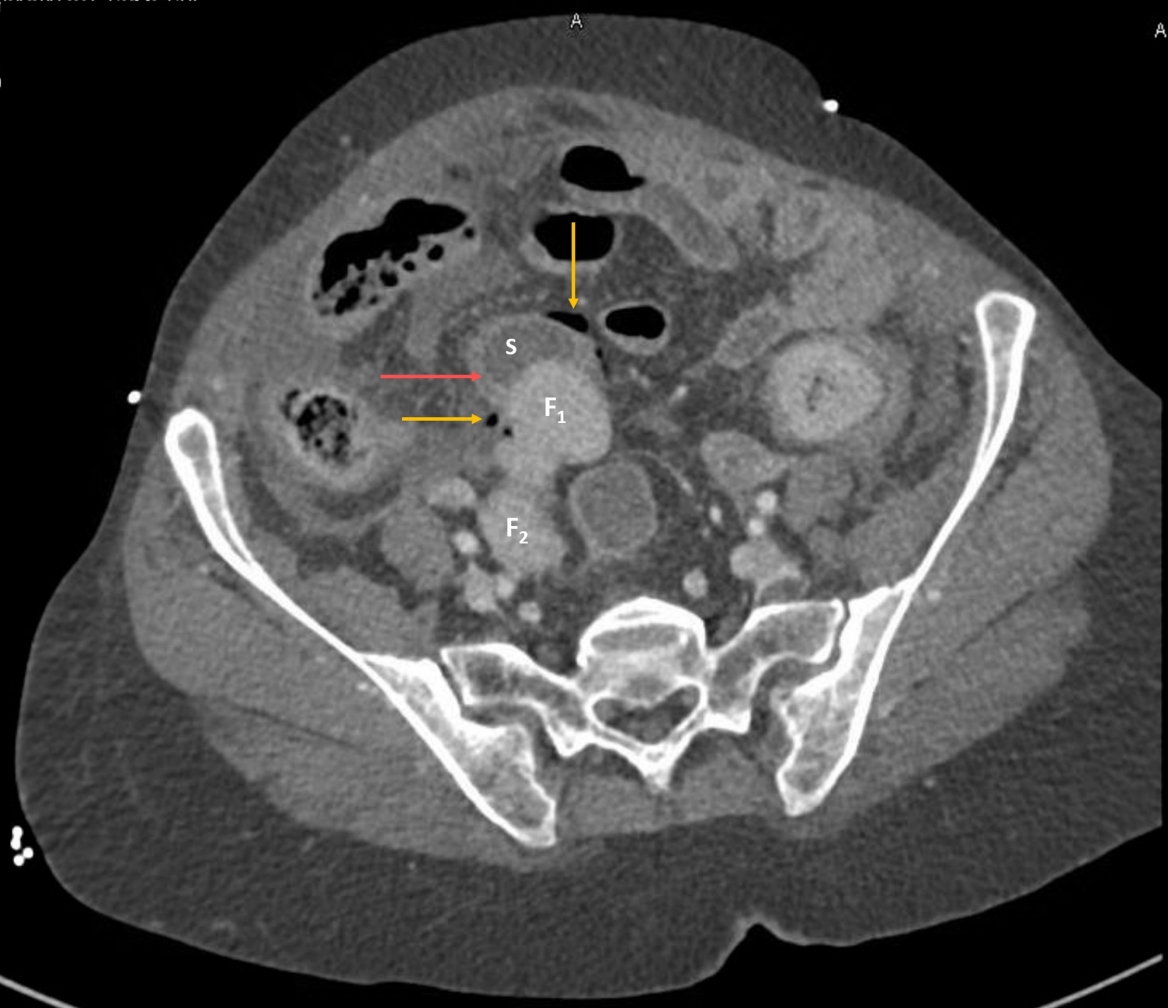
Contrast-enhanced CT scan of the abdomen, axial view F_1_ and F_2_ are faecalomas. S shows sigmoid with the bowel wall shown with red arrow. Fecaloma F_1_ is seen disrupting the sigmoid lumen continuity causing extraluminal free air (marked with yellow arrows) adjacent to the sigmoid. Fecaloma F_2_ is extraluminal and lying in the peritoneal cavity

**Figure 2:**
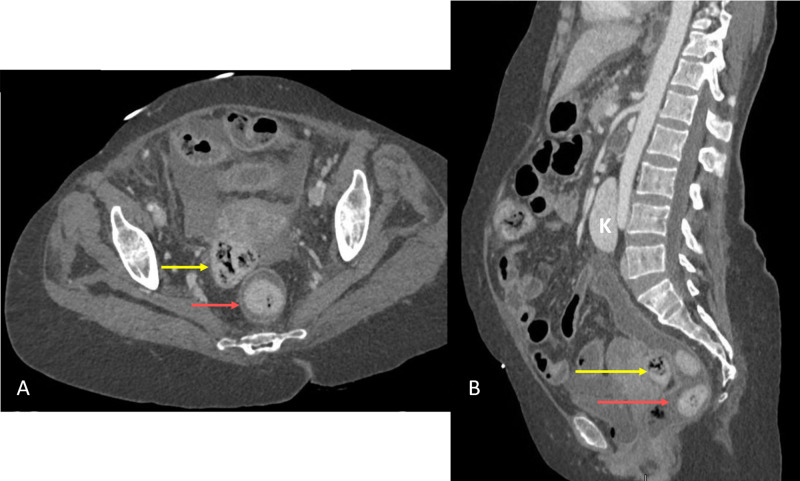
Contrast-enhanced CT scan of the abdomen, composite view (A) shows the axial view and (B) shows the sagittal view. Red arrows in (A) and (B) show intraluminal fecalomas in the rectum. Yellow arrows show extraluminal faecal ball in the peritoneal cavity in the Pouch of Douglas. Isthmus of the horseshoe kidney (K) was connecting the lower poles and is seen overlying the aorta

**Figure 3:**
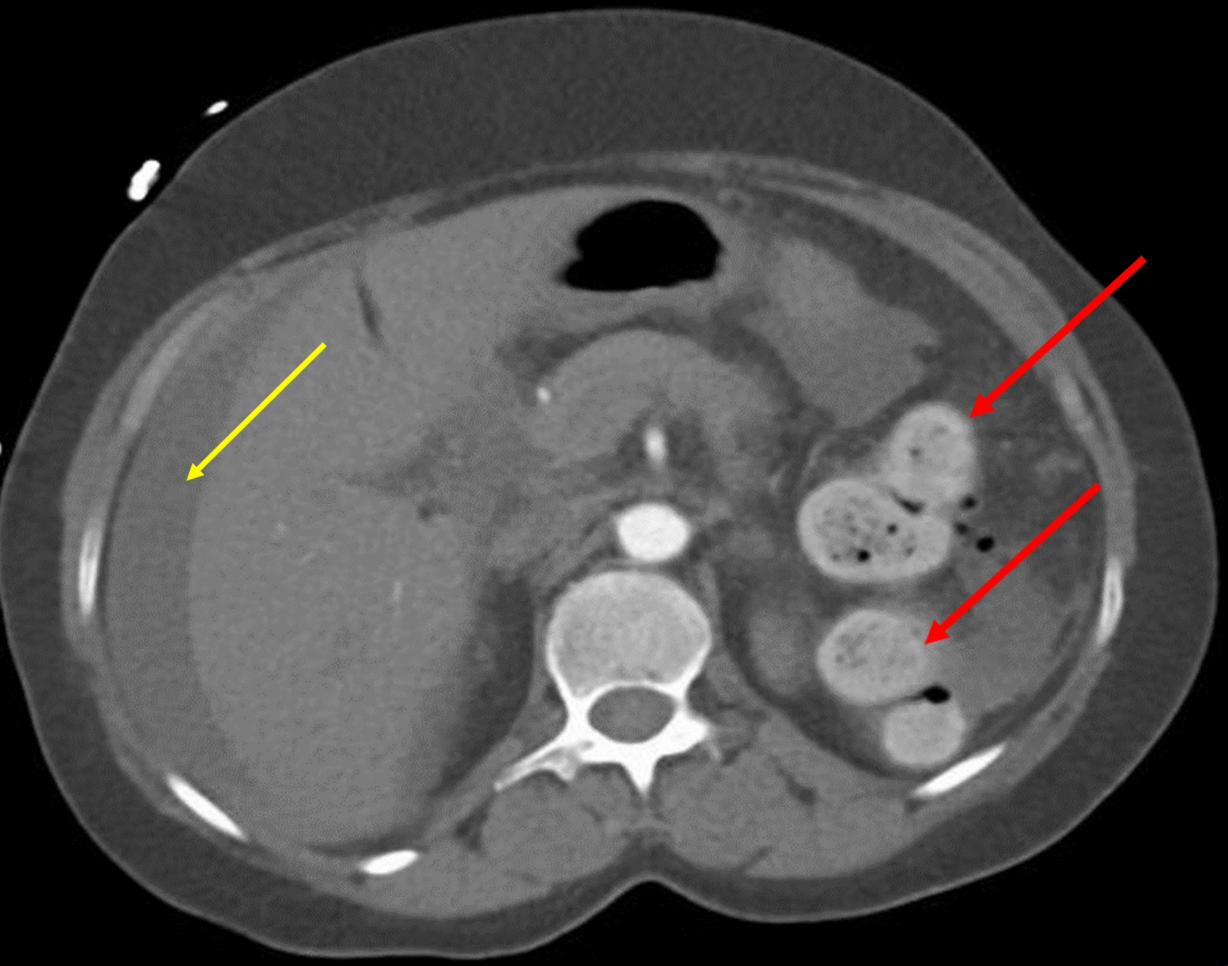
Non-enhanced CT scan of the abdomen, axial view Left colon loaded with fecal matter and indicated by red arrows. Free fluid in the peritoneal cavity is shown with yellow arrow

Chest X-ray showed no pneumoperitoneum. A contrast-enhanced CT scan showed multiple extraluminal air foci in the lower abdomen adjacent to the sigmoid colon (S) with a fecal ball (F_1_) breaching the lumen of the sigmoid (Figure 1). Multiple extraluminal fecalomas were noted in the peritoneal cavity (Figures 1, 2A, 2B). There was fecal loading of the rest of the colon, fluid in the peritoneal cavity, and mesenteric stranding and thickening in the lower abdomen (Figures 1, 2A, 2B, 3). There was mural thickening of small and large bowel loops involving descending and sigmoid colon down to the rectum. A horseshoe kidney with an isthmus connecting the lower poles was observed (Figure 2B). The aorta and its major branches showed good opacification and no definite thrombus or occlusion could be demonstrated.

Mural thickening of the left colon along with discontinuity of sigmoid lumen, pneumoperitoneum, and extraluminal fecalomas in peritoneal cavity strongly suggested perforated viscus with the possible site of perforation in the sigmoid colon.

Differential diagnosis

The differentials were perforated viscus, acute mesenteric ischemia, ischemic colitis, acute pancreatitis, and urosepsis. A normal serum amylase and lipase ruled out acute pancreatitis. Urine dipstick failed to show any signs of urinary infection but a septic workup including urine and blood cultures were sent. Initial chest X-Ray failed to show any pneumoperitoneum and the patient did not show any rigidity of the abdomen probably due to muted immune response owing to long term steroid and azathioprine use. But the possibility of bowel perforation or an ischemic event still topped the priority list amongst the differentials and prompted the CT scan which suggested SP of the sigmoid.

Initial management and surgery

As she was in septic shock, resuscitation was initiated with intravenous fluids, oxygen inhalation, broad-spectrum antibiotics and later she was started on inotropes. Her American Society of Anesthesiologists score was III E. She underwent exploratory laparotomy through a midline laparotomy incision. There was pus & feculent fluid in all the peritoneal quadrants. We found a single large perforation of 3-4 cm size at the rectosigmoid junction on the antimesenteric side. There were multiple hard fecal balls of 3-5 cm size in the peritoneal cavity corresponding to the size of the perforation and confirming the diagnosis of SP. Due to the fecal contamination of the peritoneal cavity, a history of long term steroid use, low hemoglobin, and the patient being on inotropes, it was decided to perform only a damage-control surgery with the diversion of the colon with a stoma rather than an anastomosis. Abdominal lavage was done with a copious amount of saline and a Hartmann’s procedure was performed. The abdomen was closed in layers in a tension-free manner after leaving two intraperitoneal drains.

Postoperative course

Histopathological examination of the resected sigmoid segment showed colonic perforation with surrounding inflammation, severe acute serositis, and calcified material deposition. Postoperatively, the patient developed ventilator-associated pneumonia and she was treated with antibiotics. She also developed SLE flare up and severe microangiopathic hemolytic anemia. She had a drop in hemoglobin and received multiple units of packed red blood cells after ruling out postoperative hemorrhage by a repeat CT scan. She also had plasmapheresis and intravenous immunoglobulins. The polymerase chain reaction test for cytomegalovirus (CMV) was positive. She was started on methylprednisolone 25 mg twice daily for SLE. On postoperative day 25, the patient developed fascial dehiscence which was closed with retention sutures. The steroids were tapered down.

The patient had a long-complicated stay in the hospital. After her second surgery, the patient was shifted to medical care due to SLE flare-up and recurrent chest infections. Eventually, she required mechanical ventilation. She developed abdominal collections which were drained through multiple image-guided aspirations and drainage. Later, she developed meningoencephalitis and septic shock with a worsening of renal parameters which mandated hemodialysis. But her condition worsened and eventually, she died due to multiorgan failure after seven months of her initial presentation.

## Discussion

SP is a rare cause of peritonitis. It was defined by Grinvalsky and Bowerman in 1959 as perforation of the bowel resulting from ischemic necrosis due to pressure on the colonic wall [6]. Maurer et al. described criteria to diagnose SP which are [7]:

(1) The colonic perforation is round or ovoid, exceeds 1 cm in diameter, and lies on the antimesenteric border,

(2) Fecalomas (stool balls) are present within the colon, protruding through the perforation site or lying within the abdominal cavity, and

(3) Pressure necrosis or ulcer and chronic inflammatory reaction around the perforation site are present microscopically.

The CT scan images, intraoperative findings, and specimen histopathology of our patient confirmed the presence of all three criteria thus confirming the diagnosis.

The key etiological factor responsible for SP is constipation. SP is also associated with certain drugs like opioids, non-steroidal anti-inflammatory drugs (NSAIDs), tricyclic antidepressants (amitriptyline), methadone, and verapamil [8].

Intestinal perforation has been described in SLE patients on immunosuppressive medications [4,5]. In SLE, bowel perforation occurs because of lupus mesenteric vasculitis, which causes thrombosis of vessels resulting in infarction of the bowel wall and perforation, especially of the small bowel [5]. Another proposed mechanism is due to steroid use in SLE resulting in an immunocompromised state leading to CMV infection leading to ulceration of the bowel wall and perforation [9].

However, in our case, bowel perforation occurred due to pressure necrosis of bowel induced by fecalomas. Fecalomas are hard masses of fecal matter which usually develop in patients who have chronic constipation. It usually occurs in the rectosigmoid region where feces are more dry and inspissated. This portion is also more likely to perforate as it is a watershed area when considering bowel vascularity. These fecalomas exert constant pressure on the bowel wall exceeding capillary pressure which leads to ulceration and perforation at the antimesenteric border of the bowel [6,7].

The diagnosis of bowel perforation is based on clinical history and examination. Imaging such as chest x-ray may miss bowel perforation in up to 30% of the cases. In such scenarios, a CT scan not only aids in diagnosis but also provides a clue to the site of perforation [7]. Features suggestive of SP on CT scan include discontinuity of the bowel wall, presence of fecal material either protruding through the colonic wall or lying free within the peritoneal cavity and extraluminal air [10]. Our patient had free air near the sigmoid, free fluid in the peritoneal cavity and the colon was loaded with fecal matter.

This patient requires urgent surgical intervention to control the source of sepsis, as it can be life-threatening in an immunocompromised patient. The surgical options are either a damage-control surgery like Hartmann's procedure or resection of the sigmoid colon with anastomosis [1,7]. Our patient was immunocompromised and hemodynamically unstable, so Hartmann's procedure was performed.

Although our patient recovered from sepsis, she had a complicated postoperative course which was due to her immunosuppressive medications and disease status. She had fascial dehiscence which was attributed to steroids. She underwent closure of the dehiscence with retention sutures. Following this event, a multidisciplinary meeting was arranged, and it was decided to taper down the dose of steroids. She also developed intrabdominal collections detected on CT scan which were drained percutaneously under image guidance. Unfortunately, the patient had a tumbling hospital course as she developed septic shock due to chest infection which progressed to multiorgan failure, and eventually, she died.

Although, we immediately intervened surgically for bowel perforation in our case and she did recover from sepsis initially, but later, she developed repeated chest infections due to her immunocompromised state. A multidisciplinary team including physicians, intensivists, surgeons and trained staff may improve the outcome in such cases.

## Conclusions

SP is a rare cause of colon perforation that carries a worse prognosis especially if the patients are immunocompromised. Even though stercoral perforation has not been reported in SLE yet, a combination of vasculitis, CMV infection, and pressure-induced ischemic necrosis due to hard fecalomas in chronic constipation are believed to be the probable causes. Plain X-Ray showing a pneumoperitoneum should warrant a surgical exploration.

The key to successfully managing such cases is early diagnosis, aggressive resuscitation, antibiotics, and prompt surgical intervention. SLE flare-up requiring high doses of steroids and bowel perforation creates a challenging management scenario during the postoperative period delaying wound healing and making the patient susceptible to overwhelming infections. A multidisciplinary approach is often helpful and recommended in such cases.
